# Exploring the Nature and Impact of Nurse Workload on Care: A Qualitative Study in Health Facilities in a Resource‐Limited Municipality

**DOI:** 10.1155/jonm/6684285

**Published:** 2026-08-16

**Authors:** Felix Kwasi Nyande, Kennedy Diema Konlan, Cynthia Tetteh, Priscilla Kumi, Confidence Alorse Atakro, Samuel Obeng, Patience Zottor, Derick Boadu Adu-Amoah, Gloria Owusuaa Amoah, Milipaak Japiong

**Affiliations:** ^1^ Department of Nursing, School of Nursing and Midwifery, University of Health and Allied Health Sciences, Ho, Volta Region, Ghana, deakin.edu.au; ^2^ Department of Public Health Nursing, School of Nursing and Midwifery, University of Health and Allied Health Sciences, Ho, Volta Region, Ghana, deakin.edu.au; ^3^ Department of Midwifery, School of Nursing and Midwifery, University of Health and Allied Health Sciences, Ho, Volta Region, Ghana, deakin.edu.au; ^4^ School of Nursing and Midwifery, Faculty of Health and Applied Sciences, Queensland University of Technology, Brisbane, Australia, qut.edu.au

**Keywords:** nurses, patient satisfaction, patients, workload

## Abstract

**Purpose:**

The study explored the workload and perceived quality of nursing care among nurses and patients at some selected health facilities in the Ho Municipality.

**Methods:**

This study employed an exploratory qualitative research design, involving registered nurses with at least 1 year of work experience at the selected hospitals, and patients who had been admitted for at least 3 days in three selected hospitals in the Ho Municipality. A purposive sampling technique was used in recruiting the study participants, and saturation was achieved after 19 semistructured interviews, consisting of 10 nurses and nine patients. All interviews were digitally recorded with audio recorders and were transcribed verbatim by the researchers and analyzed using thematic analysis.

**Findings:**

Staffing and resource allocations, organizational policies and cultures, patient and family factors, a lack of managerial support, and workplace violence were contributing factors to the high workload. Also, physical and mental stress, compromised patient safety and quality of care, and thoughts of changing careers were impacts of the workload. Nurses reported various coping strategies, such as improvisation, communication, working collaboratively, time management, and self‐medication. Most patients expressed satisfaction with the nurses’ interpersonal and communication skills, the privacy offered during procedures, and competence and professionalism. Patients were unhappy about the lateness of medication administration and the lack of attentiveness.

**Implications for Nursing Management:**

Adequate staffing and resources, combined with periodic in‐service training, can help alleviate the burden and enhance nurses’ physical and mental wellbeing, patient safety, and the quality of their care.

## 1. Introduction

Many nurses work on the front line daily, providing care to patients while serving as a lifeline of information, encouragement, and education to family caregivers [1–3]. Workload varies widely among nursing specialties and areas of nursing practice [4]. Globally, the prevalence of workload among nurses varies widely across all healthcare needs of patients [5]. In sub‐Saharan Africa, there is a paucity of information on the prevalence of workload among nurses [5]. In Ghana, there is little information on the prevalence of high workload among nurses; however, a nurse‐to‐patient ratio of 1:9 is considerably lower than the expected ratio of 1:4 [6–8]. The lower nurse‐to‐patient ratio indicates that the workload among nurses in Ghana might be high and, consequently, may influence the quality of care. The situation of high workload level might be compounded by other factors such as the physical working environment, lack of appropriate and adequate resources and facilities, diversity of family and patients’ needs, and ineffective communication between multidisciplinary teams and among members [2, 9]. This situation is not only peculiar to Ghana, as many other countries appear to report the shortage of qualified nurses and understaffing as a problem of growing concern [10–12]. There must seem to be a direct relationship between lower staff ratios and higher workload among healthcare professionals [13].

Increased nursing workloads negatively affect both patient care and nurses’ overall wellbeing as staff stress and burnout appear to be increasing [13–15]. The influence of higher workload on nursing services mostly leads to poor‐quality care or the inability to address the needs of patients and communities in a timely and adequate manner [8, 14]. Reports of missed care, work left undone, and poor documentation of care have been reported [16] and may be associated with higher workloads, especially in resource‐limited settings [8]. The increased nursing workload is significantly associated with higher incidents of falls, medication errors, unplanned extubations, increased acquisition of infections, and decreased odds of survival and eventual patient safety [17–22]. The higher level of nurse‐to‐patient ratios also influences the attitude of nurses toward work and service delivery [5]. A higher nurse‐to‐patient ratio may lead nurses to burn out more easily and have relatively lower levels of interest in care provision [23]. Nurses experience high levels of burnout, absenteeism, turnover, and fatigue, as well as low job satisfaction due to increased workload [5]. There is a direct correlation between nurses’ workload and patient satisfaction [24]. A prolonged increase in workload without effective coping strategies affects not only the nurses’ work but also their nursing skills and the quality of care [25].

There is a growing interest in understanding the workload of nurses and its effects on health, work output, and the quality of service delivery [1]. In Ghana, although there have not been significant improvements in the nurse‐to‐patient ratios (from 1:1251 in 2012 to 1:542 in 2016), nurses are still constrained [26]. Several studies have revealed that a heavy workload has serious implications not only for nurses but also for patients seeking healthcare [27, 28]. Some studies have been conducted in Ghana to determine the workload among nurses; however, most of these studies took a quantitative nature [28–31]. The quantitative nature of these studies does not elicit an in‐depth understanding of the factors associated with nursing workload. The lack of this in‐depth understanding of the consequences of higher workloads on nurses and patient care impedes the development of tailored interventions that may mitigate the consequences. Interventions to promote efficient nursing service delivery by limiting higher workload can only be implemented when an in‐depth understanding of the relationship between higher workload and patient care outcomes is elicited. In Ghana, the need to have this in‐depth knowledge is further heightened by the increasing discrepancies in the distribution of nurses from one area to another. There is still reported geographical inequality in the distribution of nurses (both quality and quantity) across Ghana, with the majority found within urban settings, largely concentrated in the second coastal regions [32, 33]. Consequently, having geographic‐specific contextual information about the influence of workload is imperative for localized, tailored interventions that address the gap resulting from poor care rendered in these underserved areas. Additionally, there are currently no studies conducted in the Volta region to ascertain the workload and the perceived impact on nursing care. In a multicultural country like Ghana, specific cultural contexts influence work ethics and stress perception [34]. This study explored the workload and perceived quality of nursing care among nurses and patients in some selected health facilities in the Ho Municipality.

## 2. Methods

### 2.1. Design

An exploratory descriptive qualitative research design was used [35, 36]. This design allows for the early stage to elicit a novel perspective for in‐depth exploration of a concept, as findings emanating from it can serve as an impetus for further research [36]. We adhered to the Consolidated Criteria for Reporting Qualitative Research (COREQ) checklist [37].

### 2.2. Setting

Ghana operates a three‐tier health delivery system encompassing tertiary (teaching hospitals and some specialist facilities), secondary (mostly regional and district level hospitals), and primary levels (clinics, health centers, and community health planning and services—CHPS). The three levels of facilities have nurses working and provide different levels of care with a relatively robust referral system. The Volta Region is one of the 16 administrative regions of the country, with Ho as its regional capital. The region is located in the eastern corridor and borders the Republic of Togo.

The study was conducted at the Ho Teaching Hospital, the Ho Municipal Hospital, and the Ho Polyclinic, all located in the Ho Municipality of the Volta Region. These facilities were selected as study sites because they are the major health facilities in the Ho Municipality and provide a range of healthcare services to the local population within the three‐tier healthcare delivery system. All three healthcare facilities provided diverse and multiple types of services, including curative, preventive, obstetrics, gynecology, and child health services. Critical to the provision of these services in the facility are the various cadres of nurses, including specialist nurses, general nurses, preventive nurses, and auxiliary nurses. The Ho Teaching Hospital (a tertiary‐level health facility) is a referral healthcare center with a 320‐bed capacity and 650 staff. It is well positioned to deliver specialized healthcare services to the Volta Region and beyond. All the cadres of nurses in the facility are licensed to practice. The Ho Municipal Hospital (a secondary‐level health facility) has a capacity of 186 beds. The hospital has six wards: male ward, female ward, maternity ward, labor ward, children’s ward, and Neonatal Intensive Care Unit. The Ho Polyclinic (a primary level health facility) is a 20‐bed capacity hospital with a staff strength of one hundred and ninety (190).

### 2.3. Population and Sampling

The study population included registered professional nurses (10) and inpatients (9) accessing healthcare in the selected health facilities. The eligibility criteria were registered nurses who had at least 1 year of working experience. Four of the nurses had at least a bachelor’s degree in nursing/midwifery, while the remainder had a diploma in nursing/midwifery. In the Ho Teaching Hospital five, in Ho Municipal Hospital three, and in Ho Polyclinic, two nurses were selected.

For the inpatients, those who were on admission for at least 3 days were included. Four patients were selected from the Ho Teaching Hospital, three from the Ho Municipal Hospital, and two from the Ho Polyclinic. The sample was considered at the point of data saturation. For the nurses, data saturation was reached at the 10^th^ participant, while for the patients, data saturation was reached at the 9^th^ participant. The data collection and analysis were concurrent, and data saturation was determined at a point when additional interviews did not yield any additional codes, subthemes, or themes [38]. The data collection and analysis (generation of codes and subthemes) were conducted concurrently, and the sample size was informed. The purposive sampling technique was used to recruit participants [39]. The purposive sampling technique allowed for multiple variations in the sample characteristics.

### 2.4. Data Collection

Two semistructured interview guides were used, i.e., one for the nurses and the other for patients. Each of the interview guides was structured into three (3) sections. The interview guide for the nurses had Section A, which identified the sociodemographic characteristics; Section B explored the nature of the workload and reasons for the increased workload; and Section C examined the impact of workload on nurses and patients. The interview guide for patients was structured into two. Section A identifies the sociodemographic characteristics, and Section B explores patients’ satisfaction with the services provided by nurses. All the questions on the interview guide were open‐ended questions with probes and prompts that allowed for in‐depth exploration of the concepts under discussion.

Face‐to‐face in‐depth interviews were conducted with the participants. The data collection was done by nurses (research assistants) who had at least a bachelor’s degree. Each interview session lasted between 24 and 32 min. The average time for an interview with the nurses was 28 min, while that of the patients was 25 min. The research team and assistants received training in qualitative data collection, research ethics, the study tool, and methodological rigor. During data collection, the participants were informed about the purpose of the study and were recruited after written consent was obtained. In addition, the hospital authorities of the three selected facilities provided permission. Interviews were conducted in a selected room (the consulting rooms of each facility) within each health facility. All interviews were digitally recorded with smartphone devices and were transcribed verbatim.

### 2.5. Trustworthiness

Research rigor was ensured using the four aspects of trustworthiness [39]. To ensure dependability, the researchers read over the transcripts several times, and at least two of the researchers coded the data, which were discussed among other members for validation. Also, researchers, before data collection, identified and wrote down individual biases. These biases were consistently reviewed during the data analysis process. To ensure confirmability, the researchers kept memos, field observations, and decisions regarding the data, which were incorporated into the interview transcripts. The audit trail of audios, transcripts, field notes, and meetings was labeled correctly and kept by the researchers to facilitate data integrity. Transferability was accomplished through a detailed description of the methods and tools used for replication and reference. To ensure credibility, interviews were played back to participants for clarification and to ensure that their experiences were accurately captured.

### 2.6. Data Analysis

The researchers employed a thematic approach in analyzing the data. The six steps outlined for thematic analysis of qualitative data were applied using inductive techniques [40]. The researchers read the transcripts multiple times to become familiar. The researchers manually coded the transcripts, concentrating on keywords and phrases, to create an independent coding frame after each interview session. Eventually, each transcript was compared to recognize and analyze coding differences and assess intercoder reliability. An iterative approach was used to revise codes through backward and forward data assessment. Codes that were similar or interrelated were congregated into subthemes. Subthemes that focus on a specific area were then grouped into the main themes. Four themes emerged from the data analysis.

### 2.7. Ethical Consideration

Ethical clearance was obtained from the University of Health and Allied Sciences, Research Ethics Committee (UHAS‐RECA.6[21]22‐2). The study was conducted according to the guidelines of the institutional review committee and the Declaration of Helsinki on the conduct of human subject research. Each participant provided written and verbal consent to participate. Participants were also made aware that the information they provide will be kept confidential. Therefore, in the reporting and analysis of data, names were not used.

## 3. Results

### 3.1. Sociodemographic Characteristics

The sociodemographic characteristics of the participants are shown in Table 1. Of the 10 nurses, the majority (7) were female. The nurse participants’ ages ranged from 26 to 37. More than half (6) of the nurses had a diploma certificate, and four (4) had a bachelor’s degree. The majority (6) of the nurses were married. The highest rank was Senior Nursing Officer (SNO), and the lowest rank was Staff Nurse (SN). Work experience of not less than a year and not more than four (4) years was reported. The five nurses from the Ho Teaching Hospital were each from the emergency unit, critical care unit, medical unit, surgical unit, and obstetrics and gynecology unit. Those from the Ho Municipal Hospital were from the theater/surgical unit, the outpatient department, and the emergency units, while those from the Ho polyclinic were from the child health and preventive unit and the general ward.

**TABLE 1:** Sociodemographic characteristics of nurses.

Variable	Frequency	Percentage
Age		
26–30	6	60
31–35	3	30
36–40	1	10
Sex		
Male	3	30
Female	7	70
Work experience		
1 year	3	30
2 years	1	10
3 years	3	30
4 years	3	30
Rank		
Staff nurse	4	40
Senior staff nurse	2	20
Nursing officer	3	30
Senior nursing officer	1	10
Marital status		
Single	4	40
Married	6	60
Qualification		
Diploma	6	60
Degree	4	40

The patients were four (4) males and five (5) females with ages ranging from 29 to 59 years. All participants were educated, with their highest level of education being tertiary. The majority (7) of the participants were married, while only 2 had never been married. Most (7) were employed, and two (2) were unemployed. The sociodemographic characteristics of the patients are shown in Table 2. The patients from the Ho Teaching Hospital were selected from the surgical wards, medical wards, emergency wards, and obstetrics and gynecology wards. Those from the Ho Municipal Hospital were selected from the emergency wards, medical wards, and surgical wards. Finally, those from the Ho Polyclinic were selected from the maternity ward and the general ward.

**TABLE 2:** Sociodemographic characteristics of patients.

Variables	Frequency	Percentage
Age		
Less than 35 years	4	44.4
More than 35 years	5	55.6
Sex		
Male	5	55.6
Female	4	44.4
Marital status		
Single	2	22.2
Married	7	77.8
Employment status		
Employed	7	77.8
Unemployed	2	22.2
Educational status		
Primary	2	22.2
Secondary	3	33.3
Tertiary	4	44.4

### 3.2. Workload and Perceived Quality of Nursing Care Among Nurses and Patients

Using the inductive thematic data analysis approach, four themes emerged. The themes that emerged were as follows: (1) the nature and causes of increased workload, (2) the impact of workload on the perceived quality of nursing care, (3) coping strategies adopted by nurses in curbing workload, and (4) patients’ perception of nursing care.

### 3.3. Theme One: The Nature and Causes of Increased Workload

This theme explored the nature and reasons for the increased workload among nurses. The workload of nurses is aggravated by logistical and resource constraints, poor and inadequate organizational policy, patient and family demand for quality service, misunderstandings between patients and nurses, and the general lack of a supportive culture. The subthemes that emerged included (a) human and material resource constraints, (b) patient and family expectations and care demands, and (c) ineffective or unsupportive management‐organizational culture.

#### 3.3.1. Human and Material Resource Constraints

Nurses reported the unavailability or inadequate staff commensurate with patient numbers as a critical concern for the higher workload. Nurses reported feeling overwhelmed by their workload and the number of patients they were expected to care for, with some reporting nurse‐to‐patient ratios as high as 1:6 during a particular duty shift. Participants felt that the shortage of nursing staff led to a lack of continuity in patient care and decreased quality of care. They expressed frustration with the inability to provide personalized care to patients and felt that the current staffing levels did not allow them to meet patients’ needs adequately.

*“…… the staffing shortage is one of the biggest challenges we face in providing quality care to patients. On a typical shift, I might have more than six patients to care …., with very critical cases” (P1)*

*“ …. Sometimes, we might have 32 patients with just four nurses, each with their own unique needs and challenges.… I′m constantly running from one task to the next” (P3)*

Other participants reported feeling inadequately trained and supported. They felt that the training they received was insufficient to prepare them for the complex and challenging situations. The nurse preferred that training could have made them more versatile to handle high pressures and difficulties. However, among some of the participants, this was not the case.

*“…… I still don′t feel fully prepared for all the challenges I face on the job. The training I received in school was helpful, but it didn′t cover everything I needed to be an effective nurse”* (P6)

*“Once I started working, I found that the reality of the job was much more complex and demanding than I had anticipated. ……*” (P9)

Participants lacked the necessary resources to provide adequate care. They often had to work with limited or outdated/obsolete equipment, which made the job more challenging and less efficient. Participants also reported shortages of essential supplies, such as medications and dressings. These shortages led to delays in treatment and increased stress levels.

*“…… When we′re working with outdated or limited equipment, it can be frustrating and time-consuming”* (P4)

*“Sometimes, the patient comes with respiratory distress, and we can’t even adjust the beds”* (P6)

Some participants reported a lack of teamwork and collaboration. They felt that communication between team members was poor, leading to misunderstandings and errors. Participants also reported feeling isolated and unsupported by colleagues.

*“I′ve noticed that there is a lack of teamwork and collaboration among our colleagues. Communication between team members is poor”* (P3)

*“ I feel isolated and unsupported by my colleagues. I think it would be beneficial for us to have more opportunities to work together”* (P2)

#### 3.3.2. Patient and Family Expectations and Care Demands

Participants reported that patient and family demands and expectations were a major factor in the high workload. Some participants reported that patients and families often had unrealistic expectations, such as wanting immediate responses to calls and expecting nurses to perform tasks outside the scope of practice.

*“Patients and families don′t always understand what we can and cannot do. They expect us to do everything for them, even things that are outside of our job description”* (P4)

Also, some participants reported that difficult patient behaviors and attitudes were another significant contributor. Some patients were uncooperative or verbally abusive. Participants also reported that patients who were disoriented or confused required additional attention.

“*Dealing with ‘difficult’ patients can be very stressful. They can be verbally abusive or refuse to cooperate with care”* (P9)

*“I had a patient last week who was very uncooperative and verbally abusive. Every time I went into their room to provide care, she would refuse to comply with any of my instructions, spit on me, and sometimes throw the medication away…*” (P10)

Also, other participants reported that patient acuity and complexity contributed to higher workload. Patients with multiple comorbidities, chronic illnesses, or those requiring intensive care require more time and attention.

*“I had a patient who was diagnosed with diabetes, heart disease, and kidney failure. They were also on several medications that required close monitoring, and they needed daily blood sugar checks and insulin injections. On top of that, they were also dealing with some mental health issues ….”*(P8)

Participants stated that when there were too many patients to care for, they were unable to provide adequate attention. High patient turnover also added to the high workload, as they had to constantly orient new patients to the care plan and medication regimen.

*“When we have many patients to care for, it′s hard to give each patient the attention”* (P3)

*“It′s frustrating when patients are constantly coming and going, as we have to spend time orienting them to care”* (P6)

#### 3.3.3. An Ineffective or Unsupportive Management‐Organizational Culture

Participants reported that there were inadequate policies and procedures to work effectively. There were no clear guidelines regarding what tasks were their responsibility and what tasks should be delegated.

*“I′m often unsure about what my responsibilities are and what tasks should be delegated….”* (P5)

Excessive paperwork and documentation requirements added to the high workload. Nurses were required to complete numerous forms and reports, often duplicating information in electronic health records and paper‐based systems.

*“It’s very hectic to still be writing nursing notes on sheets in this 21st century…. hmm!!” (P6)*

Participants reported feeling undervalued and unappreciated for their contributions to patient care. Nurses’ expertise and experience were not recognized or respected by other members of the healthcare team—physicians and administrators.

*“I′ve noticed that there is a lack of recognition and appreciation for the contributions that nurses make”*(P1)

Nurse managers did not provide enough guidance or support, especially when it came to making difficult decisions. They often had to make decisions without clear guidance or support from their nurse in charge, which increased stress levels.

*“I had a patient who was experiencing a lot of pain and discomfort, and I felt like we needed to adjust their medication regimen. However, when I went to the in-charge, she seemed dismissive and didn′t provide any guidance or support”* (P8)

*“I had a patient who was exhibiting symptoms that were concerning, but when I brought them up to in-charge, they didn′t seem to take me seriously. ……”* (P3)

Furthermore, the nurses reported that their managers were not always accessible or approachable, which made it difficult to address issues or seek advice. This lack of support from managers also contributed to a sense of isolation and disconnect.

*“There have been times when I′ve had issues with staffing or patient care, but I didn′t feel comfortable bringing it up with my manager. Sometimes, they′re too busy to talk, or they don′t seem approachable”* (P8)

### 3.4. Theme Two: Impact of Workload on the Perceived Quality of Nursing Care

The high workload of nurses had a significant impact on the quality of care. The impact of stress on patient outcomes includes both physical and mental stress, which affects overall health, leads to poor patient care outcomes, and poses professional challenges. The subthemes that emerged were (a) increased physical and mental stress, (b) compromised patient safety and quality care, and (c) abdication of job or profession.

#### 3.4.1. Increased Physical and Mental Stress

Nurses reported feeling physically, emotionally, and psychologically exhausted and fatigued because of a high workload. Many nurses worked long hours/days without adequate breaks, which led to physical strain and exhaustion. Another challenge nurses experience is workplace injury, which increases the risk of contracting diseases. Some nurses reported experiencing musculoskeletal pain and other physical health issues.

*“I often feel physically exhausted and fatigued after a shift. We′re constantly on our feet, lifting patients, and performing strenuous tasks”* (P6)

*“ On some shifts, we are so busy that we barely have time to take a sip of water…. I have personally experienced back problems, and even developed varicose veins”* (P9)

Nurses also reported feeling emotionally exhausted and burned out due to the high workload. Dealing with high‐stress situations, managing difficult patients, supporting dying patients and families, and navigating challenging work environments influenced emotional wellbeing. Many nurses reported feeling overwhelmed, stressed, and emotionally drained, affecting their ability to provide quality patient care.

*“Dealing with patients who are sick or in pain, managing difficult family members, and navigating the complex healthcare system can be incredibly stressful”* (P4)

The workload impacted nurses’ mental health. Some nurses reported experiencing symptoms of anxiety and depression.

*“I experience anxiety and have even managed depression. It′s not just the workload, but also the emotional strain of caring for the sick and dying”* (P9)

The high workload and increasing job demand increase the risk of workplace injuries. Some nurses reported experiencing workplace injuries such as slips and falls, back injuries, and needle‐stick injuries.

“*I′ve experienced a needle-stick injury while administering medication to a patient”* (P3)

#### 3.4.2. Compromised Patient Safety and Quality of Care

Heavy workloads contributed to an increased risk of errors and mistakes. Nurses often rush through tasks or multitask to keep up with demands, which can lead to errors in medication administration, documentation, and communication. Such mistakes had serious consequences for patients, including adverse drug reactions, falls, and other complications.

*“There was a day I came for the afternoon shift, I was serving medication, and a patient called.…. but later attended to him and realized he had hypoglycemia. Hmm, RBS of 1.2mmol/L. I felt so guilty and embarrassed”* (P1)

*“One day, I came for the night shift. I had 7 cases, and my name was also first on the triaging list. Hmmmm!!! The workload was so much that I gave my patient the wrong medications because I could not even get enough time to read the treatment plan”* (P5)

Nurses often find themselves unable to complete tasks within the allotted time, leading to delayed or missed care. This could include tasks such as turning patients to prevent pressure ulcers, administering medications, or assisting with activities of daily living.

*On one afternoon shift, we had to trans out most cases to the ward to decongest. We finished the transfers around 5 p.m., and all medications that were supposed to be administered were given later* (P9)

The workload negatively affected patient satisfaction. Due to the high demands, nurses were often unable to spend enough time with each patient, leading to patient dissatisfaction. Nurses felt that patients should receive personalized care and attention, but the workload prevented them.

*“It′s frustrating when we′re unable to give each patient the individualized care they deserve” (*P3)

*“I noticed the nurses were always running around and seemed like they didn′t have enough time to spend with me”* (P13)

*“There were times when I asked for help, and the nurse seemed so busy and stressed that they were unable to attend to me”* (P11)

Patients reported feeling dissatisfied when nurses appeared to rush over, leading to a perceived decrease in the quality of care. In addition, some patients reported feeling dissatisfied when nurses were unable to communicate effectively.

*“I had some questions about my treatment, but the nurse seemed too busy to talk to me*” (P18)

Important factors that influenced nurses’ higher workload were concerns related to patient safety and care.

*“I could tell the nurse was tired, and it worried me that they might make a mistake with my medication”* (P16)

*“The nurse seemed stressed, and it made me worried that they weren′t paying enough attention to my care. ”* (P19)

Additionally, patients described feeling less supported when nurses appeared stressed or overwhelmed.

#### 3.4.3. Abdication of Job or Profession

Nurses considered leaving their current jobs or professions. Some other nurses in Ghana have also considered traveling to practice either within or outside the country, as they are strained by several challenges in practice. Other nurses believe that their career progression will require them to further into other professional areas. Some nurses had the intention of leaving the field, while others decided to remain in the job but moved away from bedside duties

*“Lecturing is next on my agenda, but it’s not because the job is difficult”* (P2)

*“I would rather read political science and be a politician in my next generation than be a nurse”* (P10)

Some other nurses preferred to travel abroad to practice their trade or just to change their professional trajectory altogether.

“*I am planning on going to study to become a nursing practitioner, …”* (P1)

### 3.5. Theme Three: Coping Strategies Adopted by Nurses in Curbing Workload

This theme described the various coping strategies that nurses adopted to manage high workload. The strategies adopted were multiple and ranged from the ability to improvise when there was an obsolete, lack, or inadequate supply of equipment and basic consumables. The subthemes included (a) improvisation, (b) effective communication and collaboration, (c) improved time management, and (d) self‐medication.

#### 3.5.1. Improvisation

The shortage of equipment and supplies was a critical issue for the nurses, and they often had to improvise. Some participants stated that they were forced to use substandard equipment, while others reported using non‐medical items to perform medical tasks.

*“Hmmm!!! Our nebulizing machine is faulty, and the tube keeps coming out of the machine. Sometimes we secure the tip of the tube with plaster before connecting it to the machine”* (P3)

*“We had to resort to using non-medical items to perform medical tasks, like using gloves or a giving set as a tourniquet”* (P5)

The lack of essential equipment forced nurses to think creatively with problem‐solving skills. Nurses had to come up with innovative solutions to address equipment shortages, such as sharing equipment or repurposing existing equipment.

*“…, because we lack side rails and appropriate equipment to restrain patients, we use gauze and bedsheets to restrain them.”* (P3)

#### 3.5.2. Effective Communication and Collaboration

Communication and collaboration were reported by nurses as a crucial coping strategy for managing various challenges. Nurses reported that effective communication with patients and families helped manage expectations. Nurses used various communication techniques, such as active listening, empathy, and clear explanations, to facilitate patient communication.

*“When I’m communicating with patients and families, I try to actively listen to what they′re saying and acknowledge their concerns. I also make sure to use clear and simple language when explaining medical procedures”* (P4)

*“I think it’s really important to involve patients and families in care. When we explain things clearly and involve them in decision-making, they feel more empowered”* (P2)

Working collaboratively with colleagues was a crucial coping strategy for managing the workload and ensuring quality patient care. Nurses reported that working collaboratively with colleagues can also help create a supportive work environment and foster a sense of community.

*“I usually seek help from my colleagues to carry heavy patients and tasks I cannot perform”* (P2)

*“My doctor advised me not to lift heavy things, but I still do with assistance from other staff”* (P7)

#### 3.5.3. Improved Time Management

Effective time management was critical for ensuring that nurses provided quality patient care. Nurses prioritize tasks and delegate to other staff when possible. This also involved the appropriate use of student nurses and rotation nurses. They are supported to assist in patient care, complementing the activities of nurses on duty.

*“… typically, we let the rotation nurse handle the stable cases, and the students take the vital signs while we focus on the critical cases”* (P3)

Multitasking and learning to work under extreme pressure were critical for nurses to be able to complete their assigned tasks.

#### 3.5.4. Self‐Medication

Self‐medication was another strategy adopted by nurses to control body pains and discomfort. Nurses used oral analgesics (e.g., paracetamol, diclofenac) to treat body aches and discomfort.

*“Sometimes when I close from work, I will be in so much pain that I cannot do my chores, so I take a painkiller to help reduce the pain”* (P5)

Participants indicated that they sometimes resorted to a combination of oral medication and ointment.

*“For me, ‘aboniki’ and paracetamol work for me. … It is so refreshing!!”* (P3)

*“I mostly take tablet diclofenac and apply medical ice when it becomes so stressful that I can feel the body pains”* (P1)

### 3.6. Theme Four: Patients’ Perception of Nursing Care

Participants had varying experiences with nursing care, with some expressing satisfaction while others expressed dissatisfaction. Under this theme, two subthemes emerged, including (a) satisfaction with nursing care and (b) dissatisfaction with nursing care.

#### 3.6.1. Satisfaction With Nursing Care

Participants described various aspects of nursing care that influenced their satisfaction, including communication and interpersonal skills, empathy and compassion, professionalism, and availability of resources. Nurses who were able to explain medical procedures, treatments, and medications in simple and understandable terms helped patients feel more informed and involved.

*“I appreciate it when nurses explain things to me in a way that I can understand”* (P13)

*“The nurse who took care of me yesterday was very patient and explained everything in a way that I could understand”* (P16)

Nurses who demonstrated empathy and provided emotional support played a crucial role. Patients appreciated nurses who were able to connect with them on a personal level, showed compassion, and provided comfort.

*“I had a really hard time coping with my diagnosis, but the nurse was there for me every step of the way. She listened to me, showed me compassion, and helped me to feel like I was not alone in this journey”* (P17)

Others reported that nurses who exhibited respectful and courteous behavior were essential for patient satisfaction.

*“When I had a concern about my care, the nurse listened to me and took me seriously. She didn′t dismiss my worries. It made me feel like my opinion mattered”* (P15)

Patients reported feeling satisfied when nurses ensured their physical privacy by closing curtains or doors during procedures or examinations.

*“I appreciate when the nurse uses screens to screen me during the procedure.”* (P12)

Some patients also felt satisfied with nurses who were able to keep their medical information confidential.

*“I felt safe sharing my medical history with the nurse because I knew she wouldn′t share it with anyone else”* (P18)

Patients appreciated it when nurses were able to answer their questions and provide explanations clearly and concisely.

*“I felt like my nurse knew what she was doing. She was able to explain things to me in a way that made sense and answered all my questions”* (P14)

*“My nurse was able to anticipate my needs before I even asked. She knew exactly what I needed and when I needed it”* (P14)

#### 3.6.2. Patients’ Dissatisfaction With Nursing Care

Patients reported delays in medication administration. Some patients reported feeling neglected or ignored when they called a nurse, and no one came promptly.

“*I had to wait for a long time to get my medicine, even though the nurse told me it would be given at a specific time”* (P15)

*“I called the nurse several times, but nobody came for a long time. I was in pain and needed help, but it felt like nobody cared”* (P15)

Other patients stated they felt nurses were unconcerned when they described their symptoms.

*“My nurse seemed distracted and didn’t listen to me when I told her about my symptoms. She just kept telling me to rest, and it would go away, but it didn’t”* (P17)

## 4. Discussion

Nurses and patients having an in‐depth knowledge of the influence of workload in resource‐limited settings is imperative to implement targeted interventions for improvement. This study provided context‐specific information on the nature and influence of workload in the delivery of health services in resource‐constrained settings. This is particularly important because of the disproportionate distribution of nurses across the country, with the majority residing in the urban southern section of Ghana [41, 42]. The inadequate number of nurses was identified as a critical factor influencing nursing workload. This may be attributable to a high rate of nurse migration in search of greener pastures or to the relatively low rate of employment in the public sector in Ghana [43]. Even though in recent years the number of nursing students and training institutions has increased, there is a considerable number of low levels of employment within health facilities, especially in rural and other sections of the country [44, 45]. Consequently, governments must implement integrated policies to recruit and retain nurses in low‐resource areas by addressing critical staff concerns, improving service conditions, and remuneration [31]. The government, which is the highest employer in Ghana, is bedeviled by inadequate resources to employ nurses, even though training schools over the years have increased enrolment and the number of nurses trained per annum, warranting the need for private facilities [46]. Adequate staffing levels, tailored recruitment, and promoting access to necessary equipment and resources can help reduce workload, increase job satisfaction, and improve patient outcomes.

Moreover, organizational policies and culture were critical antecedents of high workload among nurses. Nurses experienced a high workload due to inadequate policies and guidelines, resulting in role ambiguity and conflicts among healthcare teams [47]. Specifically, these findings highlight the importance of having clear policies and guidelines for nurses to work effectively. Furthermore, most nurses reported that patient and family demands and expectations, and lack of managerial support, were contributing factors to high workload. In Africa, where traditional relationships are critical, family demands of care can sometimes be a reason for a higher workload [2, 48]. This warrants the continued need to have improved communication and relationships with patients and families. Hospital managers need to have improved human resources training by retraining, especially nurse managers, who are immediate supervisors. In addition, just as in this current study, in previous studies, patient and family demands and expectations were a major source of stress for nurses [49, 50]. These findings highlight the importance of addressing patient and family expectations in the healthcare system by ensuring cultural sensitivity in understanding the impact on nursing workload and job satisfaction. Further research may explore the consequential influence of patients’ expectations and the depth of influence on the nurse’s ability to perform these duties and manage stress. Most nurses reported that nurse managers were not always accessible or approachable. In other countries, nurses reported that workload and job dissatisfaction were significantly influenced by poor managerial support and ineffective leadership [51, 52]. Consequently, supervisors must prioritize open dialogue and communication between themselves and subordinates to improve care outcomes.

The findings of this study demonstrated that nurses’ physical health and mental health were negatively influenced by high workload. Many nurses felt overburdened, stressed out, and emotionally strained. Nurses reported that the high workload was related to physical health problems, which included migraines, muscle, back, and joint pain, hypertension, and duodenal ulcers [53, 54]. This highlights the need for interventions to address high workload and promote wellbeing. Heavy workload increases the risk of mistakes and errors in patient care. Higher workload of the nurses hurts the safety of the patients and the standard of care [21]. Heavy workloads among nurses led to mistakes in documentation, poor patient care, and reduced productivity [2]. It is critical to acknowledge that a heavy workload can have serious consequences for nurses and patients. Therefore, healthcare organizations need to implement strategies to address the high workload among nurses. The consideration of changing careers was another consequence of the higher workload among nurses. Higher workload was one of the main reasons that nurses considered leaving the job and the country [55, 56].

Effective communication was another coping method. Good communication with patients and families enables nurses to manage patients’ expectations. Effective communication with patients and their families is crucial for nurses to provide quality care [57, 58]. These findings highlight the importance of effective communication in nursing practice and the need for nurses to develop communication skills to cope with workload and provide quality care. Nurses alleviate workload by assigning tasks to other employees and ensuring efficient time management. Efficient time management is a better method to mitigate higher workload and lower stress levels among nurses in lower‐resource settings [59, 60]. Consequently, nurses need to prioritize tasks, delegate responsibilities when appropriate, and use time wisely to avoid burnout to ensure positive patient outcomes. In the current study, some nurses used analgesics, mainly NSAIDs (as self‐medication), to ease minor pain. This is not peculiar, as it was reported that healthcare workers, including nurses, resort to taking medications to relieve pain [61, 62]. The use of analgesics such as NSAIDs to manage pain can be effective in the short term, but it can also lead to complications, such as gastrointestinal problems, kidney damage, and cardiovascular diseases [63, 64]. Therefore, nurses need to consult with a healthcare provider before using any medications, even over‐the‐counter ones. Nurses reported that working collaboratively with colleagues can also help create a supportive work environment. This is because previous studies demonstrated that support from colleagues was a strategy adopted by most nurses to cope with a higher workload [65]. Healthcare facilities should prioritize strategies that encourage collaboration and teamwork, such as interprofessional education and team‐building exercises, to enhance the effectiveness and efficiency of care provision.

Patients expressed satisfaction when nurses protected their physical privacy during procedures or examinations by drawing drapes, closing doors, and providing screens. Patients value privacy during hospitalization and expect healthcare providers to respect and ensure privacy always [66–68]. The provision of this essential service (privacy and confidentiality) can only be achieved if nurses are not overburdened by a higher workload. Protecting patient privacy is an important aspect of nursing care that can contribute to higher levels of patient satisfaction—a critical component of quality care. Patients were more satisfied with nursing care when they perceived the nurses to be competent and professional [69, 70], including the provision of privacy and ensuring confidentiality. Nursing education and training programs should emphasize the development of clinical competence and professionalism, as they have a significant impact on patient outcomes and experiences.

### 4.1. Implications of Findings

This study used a qualitative study methodology to highlight in‐depth the factors that are associated with nurse workload and its implications on the delivery of quality nursing care. Identifying the factors associated with nurse workload is critical for improving the body of knowledge in this area and helping the development of practical interventions to improve working conditions. In addition, this study was conducted in a relatively neglected area, especially in low‐resource settings where workload is mostly high, and limited resources and interventions to improve service provision are mostly described as low. Consequently, nurse workload research is critical for nurses and other policymakers to understand the conditions under which nurses work. This can also serve as a primary point to curtail the continued and sometimes unwarranted exodus of nurses to highly developed economies for greener pastures [8, 43]. Nursing education can also benefit from the study’s findings by incorporating workload management strategies into nursing curricula. By equipping nurses with the necessary skills and knowledge to manage workload effectively, they can be better prepared to meet the demands of practice [71]. Also, nursing programs should emphasize the importance of communication and interpersonal skills, time management, and problem‐solving skills. They should also provide training on the use of electronic health records to improve efficiency and reduce workload. Further research on workload management strategies and their effectiveness in improving patient outcomes and nursing satisfaction is warranted.

### 4.2. Strengths and Limitations

This study has several strengths. Even though the use of exploratory descriptive qualitative designs has inherent limitations, as it cannot produce a definitive quantity of influence, it produces textual information that gives an independent understanding, context, and meaning to the phenomenon. This design is a primary starting point to begin to consider nurse workload and resultant influence on quality care, especially in resource‐limited settings. In addition, we noted that logistical and resource constraints, unclear/inadequate organizational/practice policy, etc., and their related influence on nurses’ work in resource‐limited settings can further be explored within the context of another study using experimental or quantitative designs. Some critical limitations may be related to the fact that this study did not involve the administrative managers of the facilities where the study was conducted. Consequently, valuable input was not incorporated. Future research studies may consider including this cadre to give a diverse perspective on the influence of nurses’ workload on service delivery. Additionally, an in‐depth understanding could have been elicited if focus group discussions as a data collection method had been adopted. Regardless of this limitation, the study gives a primary focus to determine the influence of nursing workload on the delivery of quality nursing services in resource‐limited Ghanaian settings.

## 5. Conclusion

This study highlights the importance of addressing workload concerns to improve nurses’ physical and mental health, patient safety, and the quality of care. Adequate staffing and resources, along with periodic in‐service training, can help alleviate the workload burden of nurses in resource‐limited settings. Patients value nurses’ interpersonal and communication skills, competence, and professionalism, and the appropriate level of privacy during procedures. Consequently, it is important to have the required number of staff to be able to provide these professional nursing services to patients, warranting improvement in the nursing‐to‐patient ratio. Nursing managers and leaders should work toward implementing policies and procedures that prioritize staffing and resource allocation, effective communication, and teamwork. Nurses themselves can also take action to manage workload by utilizing creative problem‐solving skills, effective communication, time management techniques, and teamwork. Further research on workload management strategies and their effectiveness in improving patient outcomes and satisfaction is warranted.

## Author Contributions

Felix Kwasi Nyande, Kennedy Diema Konlan, Cynthia Tetteh, Priscilla Kumi, Confidence Alorse Atakro, Samuel Obeng, Patience Zottor, Derick Boadu Adu‐Amoah, Gloria Owusuaa Amoah, and Milipaak Japiong were involved in conceptualization. Felix Kwasi Nyande, Cynthia Tetteh, and Kennedy Diema Konlan were involved in planning. Confidence Alorse Atakro, Samuel Obeng, Patience Zottor, Derick Boadu Adu‐Amoah, and Gloria Owusuaa Amoah collected the data. Felix Kwasi Nyande, Cynthia Tetteh, and Kennedy Diema Konlan performed analysis. Felix Kwasi Nyande, Kennedy Diema Konlan, Priscilla Kumi, Confidence Alorse Atakro, Samuel Obeng, Patience Zottor, Derick Boadu Adu‐Amoah, Gloria Owusuaa Amoah, and Milipaak Japiong prepared the initial draft of the manuscript, and Kennedy Diema Konlan reviewed the manuscript for significant intellectual inclusion.

## Funding

No funding was received for this research.

## Disclosure

All authors read and approved the final manuscript.

## Ethics Statement

Ethical clearance was obtained from the University of Health and Allied Sciences, Research Ethics Committee (UHAS‐RECA.6[21]22‐2). The study was conducted according to the guidelines of the institutional review committee and the Declaration of Helsinki on the conduct of human subject research. Each participant provided written and verbal consent to participate. Participants were also made aware that the information they provide will be kept confidential. Therefore, in the reporting and analysis of data, names were not used.

## Consent

The authors have nothing to report.

## Conflicts of Interest

The authors declare no conflicts of interest.

## Data Availability

All data analyzed during this study are included in this published article. Detailed audio recordings and transcripts of the entire manuscript can be obtained from the corresponding author upon reasonable request.
